# Neuromuscular characteristics of front and back legs in junior fencers

**DOI:** 10.1007/s00221-022-06403-w

**Published:** 2022-06-30

**Authors:** Kohei Watanabe, Akane Yoshimura, Aleš Holobar, Daichi Yamashita, Shun Kunugi, Tetsuya Hirono

**Affiliations:** 1 Laboratory of Neuromuscular Biomechanics, School of Health and Sport Sciences, Chukyo University, Tokodachi, Kaizu-cho, Toyota, 470-0093 Japan; 2 Faculty of Education and Integrated Arts and Sciences, Waseda University, Tokyo, Japan; 3 Faculty of Electrical Engineering and Computer Science, University of Maribor, Maribor, Slovenia; 4 Department of Sport Science, Japan Institute of Sport Sciences, Kita-ku, Tokyo, Japan; 5 Center for General Education, Aichi Institute of Technology, Toyota, Japan; 6 Research Fellow of Japan Society for the Promotion of Science, Tokyo, Japan

**Keywords:** Motor unit, Youth athletes, Quadriceps, Vertical jump, High-density surface electromyography

## Abstract

In elite fencers, muscle strength and muscle mass of the front leg (FL) are greater than those of the back leg (BL) due to characteristic physiological and biomechanical demands placed on each leg during fencing. However, the development of laterality in their neural and muscular components is not well-understood. The present study investigated neuromuscular characteristics of FL and BL in junior fencers. Nineteen junior fencers performed neuromuscular performance tests for FL and BL, separately. There were no significant differences in the isometric knee extension strength (MVC), unilateral vertical jump (UVJ), vastus lateralis muscle thickness (MT), or motor unit firing rate of the vastus lateralis muscle (MUFR) between FL and BL (*p* > 0.05). In subgroup analyses, a significantly greater MUFR in FL than BL was noted only in fencers with > 3 years of fencing experience, and significantly greater UVJ in FL than BL was observed solely in fencers with < 3 years of fencing experience (*p* < 0.05). Strong positive correlations between FL and BL were identified in MVC, MT, and MUFR in fencers with > 3 years of fencing experience, but not in those with < 3 years of experience. These findings suggest that in junior fencers, laterality in neuromuscular performance has not manifested, whereas longer fencing experience induces fencing-dependent laterality in neural components, and laterality in dynamic muscle strength is decreased with fencing experience.

## Introduction

Fencers are representative athletes with laterality in physiological and morphological characteristics. Greater muscle mass and strength of the weapon arm and front leg (FL) compared with their opposing sides in fencers have been reported in several studies (Nystrom et al. 1990; Roi and Bianchedi 2008; Tsolakis et al. 2006). Fencers use one arm to hold the weapon and the leg on the weapon-held side maintains a forward position during the competition. A previous study that investigated neuromuscular coordination of leg muscles during fencing attack suggested that knee and hip extensor muscles of FL and the back leg (BL) contribute to braking actions with eccentric contraction and propulsive action with concentric contraction, respectively (Guilhem et al. 2014). These functional differences would induce physiological and morphological laterality between FL and BL in fencers (Nystrom et al. 1990; Roi and Bianchedi 2008; Tsolakis et al. 2006). Most of these studies focused on elite fencers with a longer history of fencing competition, such as an 8-year fencing training history (Tsolakis and Vagenas 2010), while little is known about the physiological and morphological characteristics of junior fencers. Tsolakis et al. (2006) reported that asymmetries in leg muscle morphology were observed in 14- to 17-year-olds with a 4.4-year fencing training history, but not in 10- to 13-year-olds with a 2.2-year history (Tsolakis et al. 2006). Based on this, we consider that morphological laterality in fencers is elicited by more than 2 or 3 years of fencing training in junior fencers. However, the process of this adaptation in junior fencers is not fully understood due to a lack of detailed data on neuromuscular performance.

Muscle strength, which is a major indicator of neuromuscular performance, is mainly determined by variables in peripheral muscles and the central nervous system (Gabriel et al. 2006; Moritani and deVries 1979; Sale 1988). These muscular and neural components show different time-courses in training induced-adaptations in young and older adults (Kamen 2005; Moritani and deVries 1980). Also, their contributions to muscle strength may change age-dependently (Watanabe et al. 2016; Watanabe et al. 2018a, b). Reports on trainability or contributions to muscle strength of these two components in children or junior athletes are controversial (Bouchant et al. 2011). Younger adults have been reported to exhibit greater trainability in neural components (Lloyd et al. 2014). This concept is supported by previous studies that reported greater muscle strength relative to muscle size in young adults than children (Kanehisa et al. 1994) or improvements of muscle strength with increased muscle activation and without significant morphological adaptations following resistance training intervention (Ramsay et al. 1990). However, another study found no significant changes in the ratio between muscle strength and muscle mass among younger adults before and after puberty and adults (O'Brien et al. 2010), suggesting that no age-dependent difference in trainability of muscular and neural components in younger adults exists. Separate analysis of muscular and neural components would help to understand the process of development of neuromuscular performance in younger adults, and would be useful for planning training strategies based on growth and development in younger athletes. On the other hand, difficulty in quantification of neural components has been recognized due to methodological limitations, while measurements of muscular components, such as muscle thickness or mass using ultrasonography or other medical imaging techniques, have been widely applied in related research areas. High-density surface electromyography (HDsEMG) has been developed with novel algorithms to measure motor unit properties (Farina et al. 2008; Holobar et al. 2009; Merletti et al. 2008), direct physiological parameters of neural input from the central nervous system to peripheral muscles. Because of its noninvasiveness, this methodology can be applied to various populations including children, older adults, patients, and athletes.

The present study investigated neuromuscular characteristics of FL and BL in junior fencers and the effect of the duration of fencing on them. We hypothesized that in junior fencers: (1) muscle strength and thickness are greater in FL than BL, (2) the neural component is also more developed in FL than BL, and (3) laterality of neuromuscular characteristics is influenced by the duration of fencing experience.

## Methods

### Participants

Nineteen young fencers, 14 males and 5 females, [mean ± SD: age: 15.8 ± 1.1 (14–17) years, height: 165.7 ± 8.1 cm, body mass: 57.3 ± 7.5 kg] participated in this study. The mean duration of fencing experience was 4.9 ± 3.7 years. The fencers in the present study consisted of ten foil fencers (five males and five females), four epee fencers (four males), and five sabre fencers (five males). FL and BL were determined as the leg on the weapon-held side and contralateral side, respectively. In 14 out of the 19 participants, the right leg was FL. A priori analysis of sample size for the present study was conducted using G*Power software (version 3.1, Heinrich Hein University, Dusseldorf, Germany). Referring to a previous study (Nystrom et al. 1990), power analysis using an effect size of 0.8 (large effect size), with an *α* error of 0.05, and a power of 0.80, revealed that the required sample size was 12 subjects for a comparison between limbs. Written informed consent was obtained from all participants with their guardians after providing them with a detailed explanation of the purposes, potential benefits, and risks associated with participation. This study was approved by the Research Ethics Committee of Chukyo University (2021-13).

### Measurements

Participants performed isometric contraction of unilateral knee extension for both right and left legs on a dynamometer (Takei Scientific Instruments Co., Ltd., Niigata, Japan). Based on the force measured by a force transducer (LU-100KSE; Kyowa Electronic Instruments, Tokyo, Japan) fixed at the distal part of the shank segment and the distance between the transducer and estimated knee joint center as the moment arm, knee joint extension torque was calculated. Maximal voluntary contraction (MVC) and submaximal contractions with the knee/hip angle at 90° were measured following a warm-up session. Two MVCs were performed with a 2-min rest interval between them for right and left legs, respectively. An MVC trial included a gradual increase in the knee extension force to maximum effort in 2–3 s, and the plateau phase at maximum effort was maintained for 2–3 s with a verbal count given at 1-s intervals. Force signal is detected by force amplifier (TSA-110, Takei Scientific Instruments Co., Ltd., Niigata, Japan) with 190 Hz of sampling rate. Peak force measured in this amplifier system during MVC trial was determined as MVC force.

**Fig. 1:**
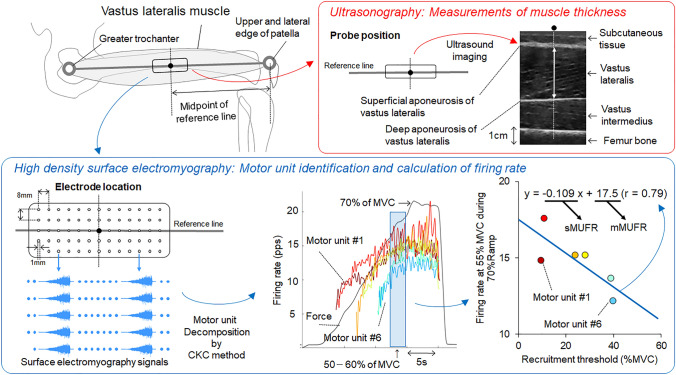
Experimental set up for recording high-density surface electromyography and ultrasonography from vastus lateralis muscle. Electrode location of high-density surface electromyography and process of calculations of motor unit firing properties (bottom). Association between motor unit firing rate and recruitment threshold for back leg in a fencer and calculation of the slope (sMUFR) and intercept (mMUFR) for the detected motor units (right bottom). Probe position of ultrasonography and longitudinal ultrasound image for measuring muscle thickness of vastus lateralis muscle. The distance between superficial and deep aponeurosis of vastus lateralis was measured at the center of probe. *MVC* maximal voluntary contraction

Three different ramp contractions were applied as submaximal contraction. Individual ramp contractions consisted of a 15, 17, and 14-s increasing ramp phase from the baseline to 30, 50, and 70% of MVC force levels with an approximately 2, 3, and 5% of MVC/s rate of force increase and 15, 10, and 5-s sustained holding phase at 30, 50, and 70% of MVC force levels, respectively. Participants were provided with visual feedback of target and performed force via a monitor. One successful trial was used for further analyses for each force level. Thus, participants performed at least one trial for 30, 50, and 70% of MVC force levels. If the exerted force was not correctly traced the target force line, additional trial was given for each contraction level. During ramp contractions, high-density surface electromyography (HDsEMG) signals were recorded from the vastus lateralis (VL) muscle using a semi-disposable adhesive grid of 64 electrodes (13 rows and 5 columns with one missing electrode) with a 1-mm diameter and 8-mm inter-electrode distance (GR08MM1305, OT Bioelectronica, Torino, Italy). The electrode grids were placed at the midpoint of the line between the head of the greater trochanter and upper lateral edge of the patella, and the long side of grids was aligned along the reference line (Fig. 1). A wet electrode strap (WS2, OT Bioelectronica, Torino, Italy) was placed at the knee. Monopolar surface EMG signals were recorded with a band-pass filter (10–500 Hz), amplified by a factor of 256, sampled at 2000 Hz, and converted to digital form by a 16-bit analog-to-digital converter (Sessantaquattro, OT Bioelectronica, Torino, Italy). The signal from the force transducer of the dynamometer was also recorded and synchronized with this analog-to-digital converter.

During the preparation for attaching HDsEMG electrodes, muscle thickness (MT) of the VL muscle was measured using an ultrasound device b-mode (iViz air, FUJIFILM Medical Co., Ltd.). We instructed participants to sit on a chair with the knee/hip angle at 90° during the measurement with their legs relaxed. The ultrasound probe (10–5 MHz) was placed on the skin at the center of the HDsEMG electrode grid. We took two ultrasound images from a side of legs in each participant. From a longitudinal image of ultrasonography, the distance between the superficial and deep aponeurosis of VL was measured as the muscle thickness by imaging software (ImageJ; National Institute of Health) (Watanabe et al. 2018a, b) (Fig. 1). The averaged values of muscle thickness of VL from two images measured in a leg from a participants were used for further analyses.

After the knee extension tasks, the participants performed unilateral vertical jump (UVJ) on two force platforms (Takei Scientific Instruments Co., Ltd., Niigata, Japan) to measure vertical forces of right and left legs separately. Vertical force was sampled at 1000 Hz by an A-D converter (Power Lab 16/35, AD Instruments, Melbourne, Australia). UVJ performance is useful for assessing dynamic leg strength like isokinetic force (Bishop et al. 2021; Menzel et al. 2013). Participants were instructed to push the ground vertically to jump as high as possible by one leg, and the depth of counter movement was freely chosen by each participant to adopt a comfortable posture following the trial for familiarization. Their right or left feet were placed on different platforms to measure vertical forces separately, and their hands were held at their waists to restrict arm movements during UVJ. Following familiarization and warm-up, two UVJs were performed for right or left legs, respectively. The impulse of the vertical force during the propulsive phase was calculated and used for further analysis.

### Data analysis

The highest MVC of two trials was determined as MVC of right and left legs, respectively, and used for target forces of submaximal contractions.

During submaximal contractions, individual motor units were identified from the recorded monopolar surface EMG signals by the Convolution Kernel Compensation (CKC) technique using DEMUSE software (Holobar et al. 2009; Holobar and Zazula 2004, 2008; Merletti et al. 2008). The procedures for decomposition into individual motor units used in the present study were previously and extensively validated based on HDsEMG signals from various skeletal muscles, including the VL muscle (Farina et al. 2010; Gallego et al. 2015a, b; Holobar et al. 2009; Watanabe and Holobar 2021; Watanabe et al. 2016; Yavuz et al. 2015). Based on Holobar et al. (2014), the pulse-to-noise ratio (PNR) was used as an indicator of the motor unit identification accuracy, and only motor units with PNR > 30 dB (corresponding to an accuracy of motor unit firing identification > 90%) were employed for further analysis; all other motor units were discarded (Holobar et al. 2014). We used motor unit firing properties during the ramp contraction to 70% of MVC for further analyses. To detect the motor units recruited at lower force levels during the ramp contraction to 70% of MVC, motor units were detected using the MU filter estimated from surface EMG during ramp contractions to 30 and 50% of MVC with lower ramp rates to the EMG signals recorded during the ramp contraction to 70% of MVC, as described previously (Del Vecchio et al. 2019a, b; Frančič and Holobar 2021). The previously introduced criterion of PNR > 30 dB was also applied to motor unit tracking, ensuring an accuracy of motor unit firing identification > 90% during ramp contraction to 70% of MVC. Median values of instantaneous firing rates when participants exerted forces between 50 and 60% of MVC during the ramp contraction to 70% of MVC were used for individual motor units and applied for further analysis. Motor unit firing behavior shows inter-individual characteristics at higher force levels in our previous study such as effects of aging and training intervention (Watanabe et al. 2016, 2021). On the other hand, motor unit firing rate rapidly declined during sustained phase at higher force level (Bigland-Ritchie et al. 1983; Garland and Gossen 2002). Also, some participants performed force production with large force fluctuation at over 60% of MVC during the ramp contraction to 70% of MVC. Therefore, we used this force level (50–60% of MVC) for analysis of motor unit firing properties. For the right or left leg of individual fencers, scatter plots of firing rates of individual motor units and recruitment thresholds were analyzed and linear regression was calculated between them (*y* = *ax* + *b*) (Fig. 1). Since the motor unit firing rate is strongly influenced by the recruitment threshold, and their association is negatively and linearly correlated (Parra et al. 2021), this effect should be accounted for. Therefore, the present study used the intercept of this equation (b) as an indicator of neural characteristics in individual fencers, i.e., the modified motor unit firing rate (mMUFR). The slope of this equation (a) was also used as indicator of motor unit firing properties (sMUFR).

To examine the effect of fencing experience, participants were divided into two groups: less [< 3 years (F3−)] or more [> 3 years (F3+)] than 3 years of fencing experience. As stated in Table 1, eight and ten participants were enrolled in the two groups, respectively.

**Table 1:** Characteristics of fencers with less (F3−) or more (F3+) than 3 years of fencing experience

	F3−	F3+	
	8 (5 males, 3 females)	11 (9 males, 2 females)	
	4 foil, 2 epee, 2 sabre 8 right-handed	6 foil, 2 epee, 3 sabre 7 right-handed	F3− vs F3+
Age (years)	15.6 ± 0.7	15.9 ± 1.3	n.s
Height (cm)	164.3 ± 6.2	166.8 ± 9.4	n.s
Body mass (kg)	53.9 ± 5.0	59.7 ± 8.3	n.s
Fencing experience (years)	1.1 ± 0.9	7.6 ± 2.1	*p* < 0.001
MVC (Nm/BM)			
FL	2.9 ± 0.7	3.5 ± 0.7	n.s
BL	2.8 ± 0.8	3.4 ± 0.6	n.s
MT (cm)			
FL	2.7 ± 0.5	2.7 ± 0.3	n.s
BL	2.6 ± 0.3	2.7 ± 0.4	n.s
mMUFR (pps)			
FL	19.5 ± 4.2	21.1 ± 4.6	n.s
BL	23.2 ± 9.1	18.2 ± 5.7	n.s
UVJ (N s)			
FL	14.2 ± 1.9	16.0 ± 3.3	n.s
BL	13.1 ± 1.9	15.2 ± 3.4	n.s

MVC- maximal voluntary contraction, FL- front leg, BL- back leg, MT- muscle thickness, mMUFR- modified motor unit firing rate, UVJ- unilateral vertical jump

### Statistics

All data are presented as the mean ± SD. We used non-parametric statistical tests in the present study based on the results of the Shapiro–Wilk test. MVC, MT, mMUFR, sMUFR, and UVJ were compared between FL and BL using the Wilcoxon signed rank test for all participants, F3−, and F3+. Using Spearman’s rank correlation coefficient, correlation analysis between FL and BL in MVC, MT, mMUFR, sMUFR, and UVJ was also performed in all participants, F3−, and F3+ groups, to assess their laterality. Age, height, body mass, and MVC, MT, mMUFR, sMUFR, and UVJ for FL and BL were compared between F3− and F3+ using the Wilcoxon signed rank test. Additionally, the effects of sex (male/female) and weapon types (foil/epee/sabre) were also analyzed following the above mentioned process for all participants, F3−, and F3+ groups. Effect size (ES) was calculated as correlation coefficient for each comparison (Tomczak and Tomczak 2014). Statistical analysis was performed using SPSS (version 21.0, SPSS, Tokyo, Japan), and the level of significance was set at 0.05.

## Results

Average numbers of detected motor units for the calculation of firing rates were 6.9 ± 3.5 for FL and 7.1 ± 3.0 for BL for each participant. There were no significant differences in numbers of detected motor units for the calculation of firing rates between F3− and F3+ (*p* > 0.05).

**Fig. 2:**
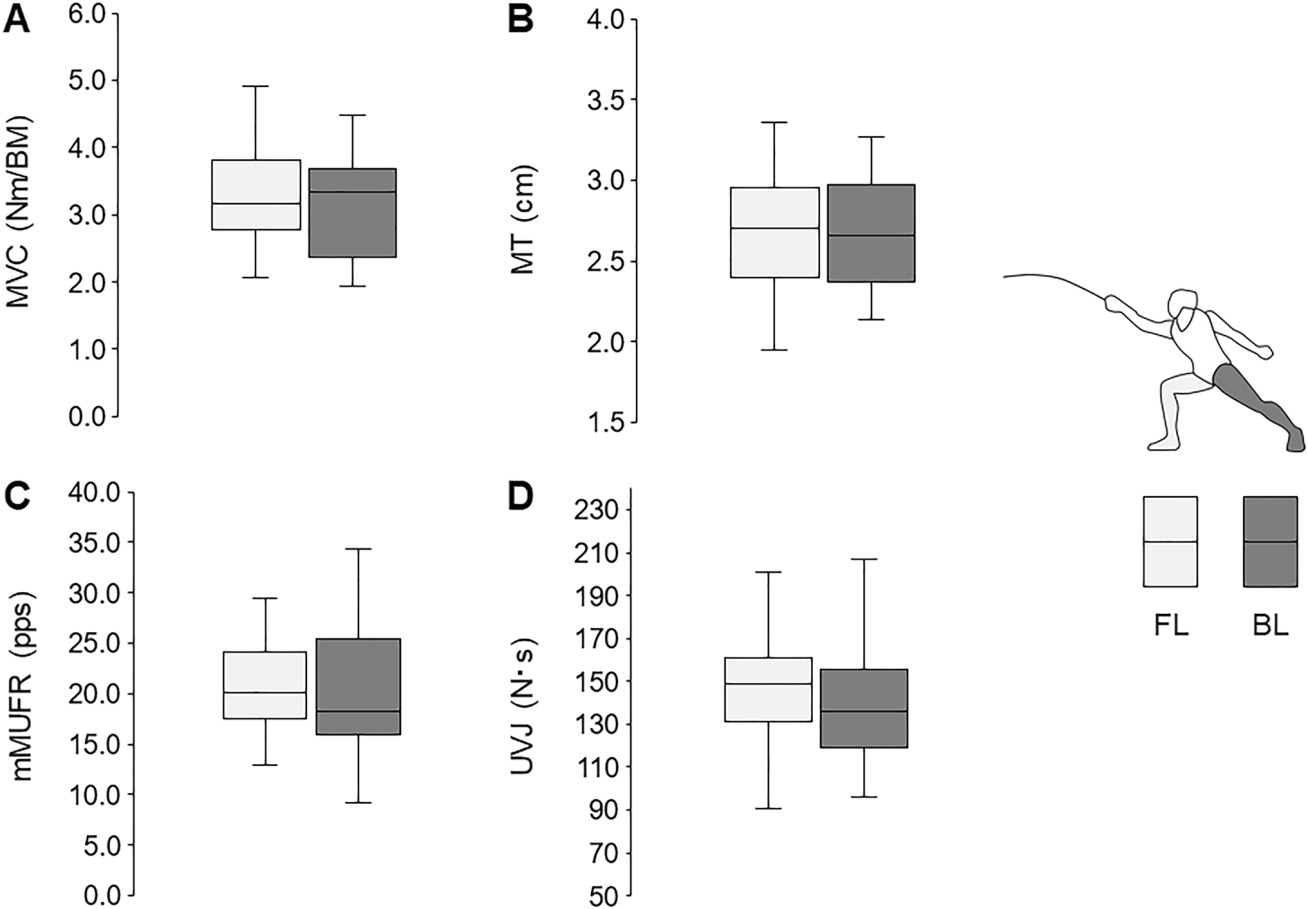
Comparisons between front leg (FL) and back leg (BL) in maximal voluntary contraction (MVC) (**A**), muscle thickness (MT) (**B**), modified motor unit firing rate (mMUFR) (**C**), and unilateral vertical jump (UVJ) (**D**) for all participants on grouping both fencers with less (F3−) and more (F3+) than 3 years of fencing experience

For all participants grouped together, no significant differences were observed in MVC, MT, mMUFR, or UVJ between FL and BL (Fig. 2A–D) (*p* > 0.05).

**Fig. 3:**
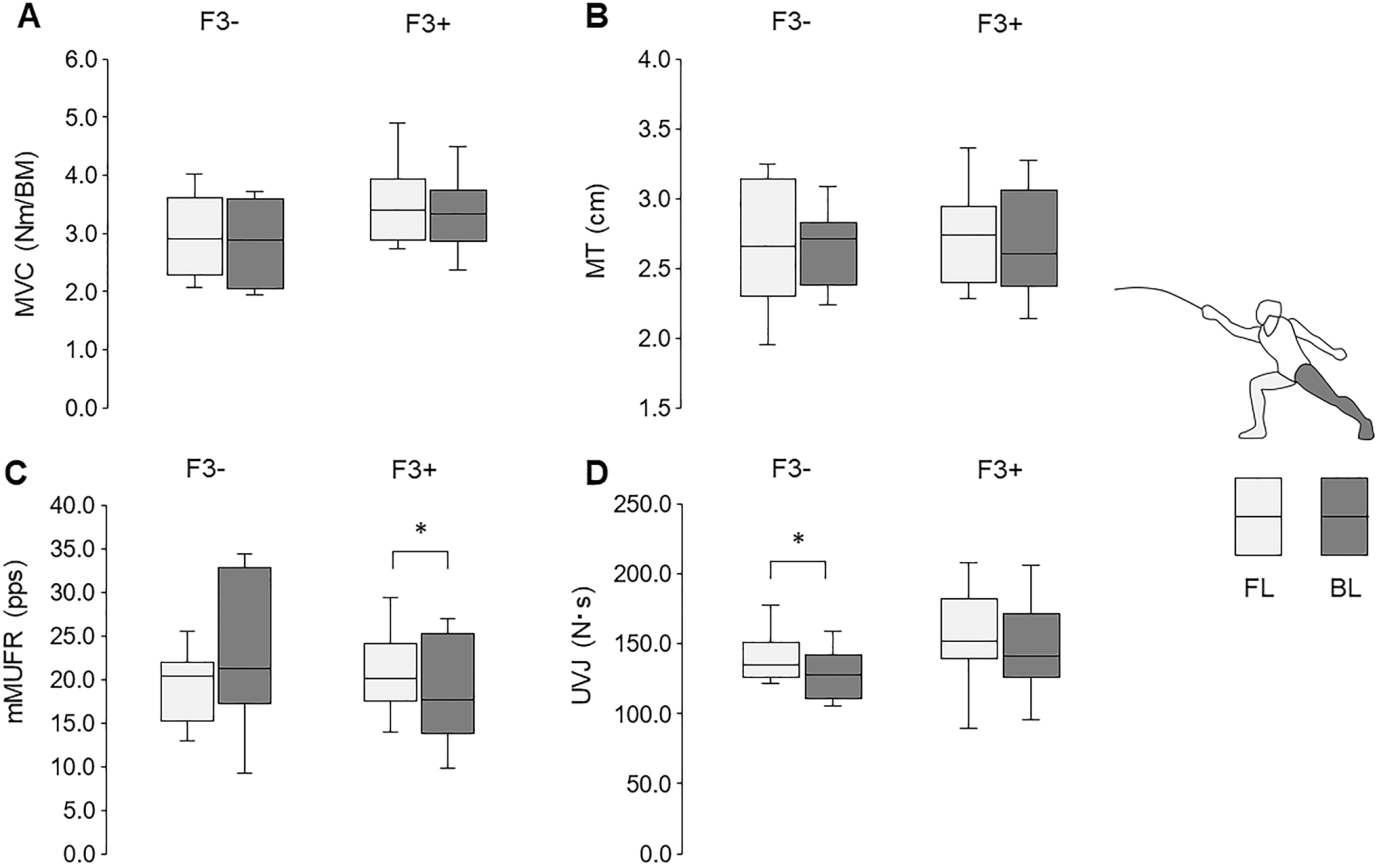
Comparisons between front leg (FL) and back leg (BL) in maximal voluntary contraction (MVC) (**A**), muscle thickness (MT) (**B**), modified motor unit firing rate (mMUFR) (**C**), and unilateral vertical jump (UVJ) (**B**) for fencers with less (F3−) and more (F3+) than 3 years of fencing experience. *p < 0.05 between FL and BL

**Fig. 4:**
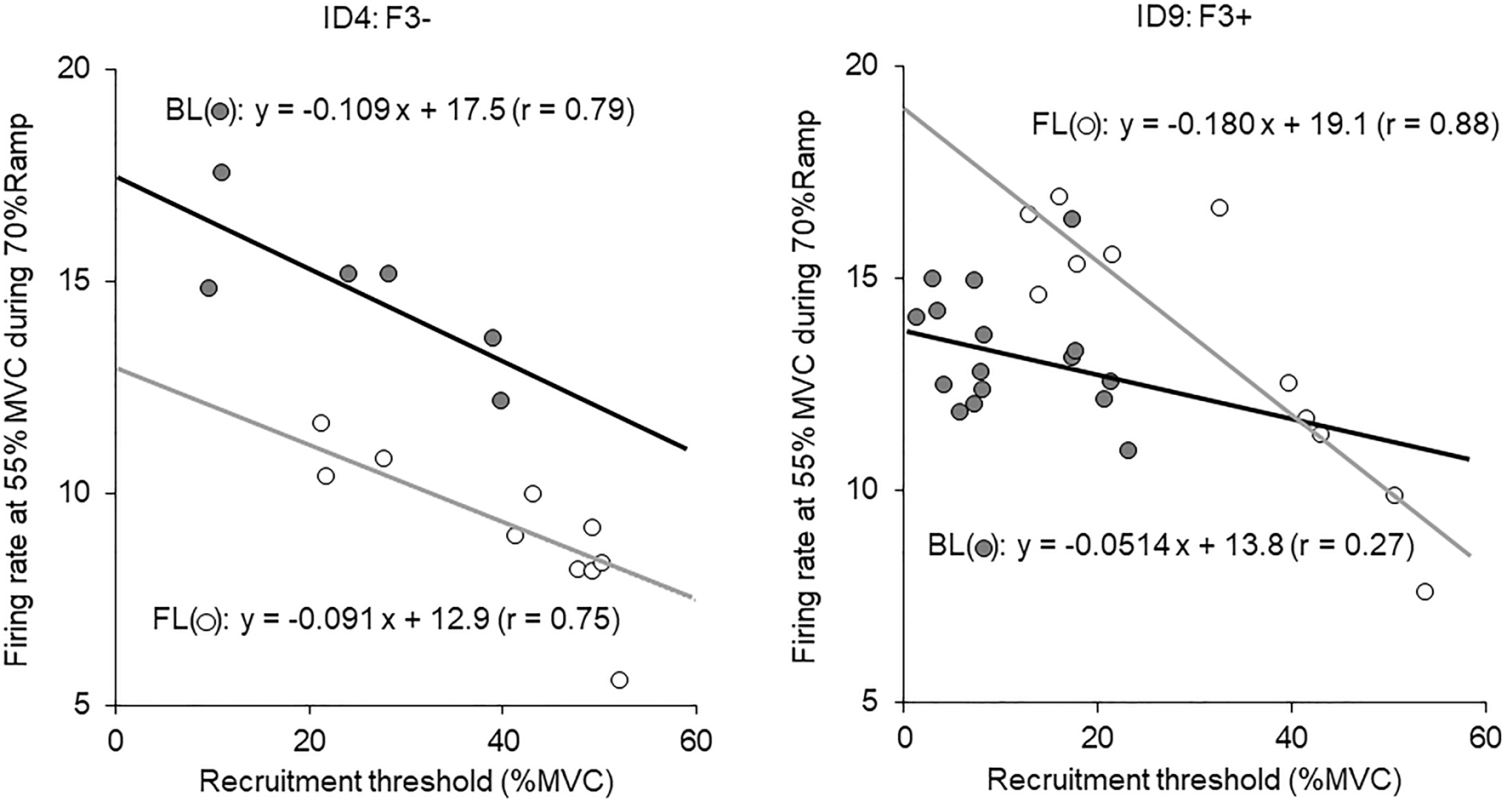
Representative data for relationships between motor unit recruitment threshold and firing rate for the fencer with less (F3−) (left) and more (F3+) (right) than 3 years of fencing experience for front (FL) (open circle) and back leg (BL) (filled circle)

There were no significant differences in age, height, body mass, MVC, MT, mMUFR, or UVJ for FL and BL between F3− and F3+ (Table 1). No significant differences in MVC or MT were noted between FL and BL for either F3− or F3+ (*p* > 0.05) (Fig. 3A, B). A significantly greater mMUFR was shown in FL compared with BL for F3+ (*p* < 0.05, ES = 0.777), but not for F3− (*p* > 0.05) (Fig. 3C). Representative data for relationship between recruitment thresholds and motor unit firing rates for FL and BL in participants from F3− and F3+ were shown in Fig. 4. UVJ in FL was greater than that in BL for F3− (*p* < 0.05, ES = 0.791), but not for F3+ (*p* > 0.05) (Fig. 3D).

**Fig. 5:**
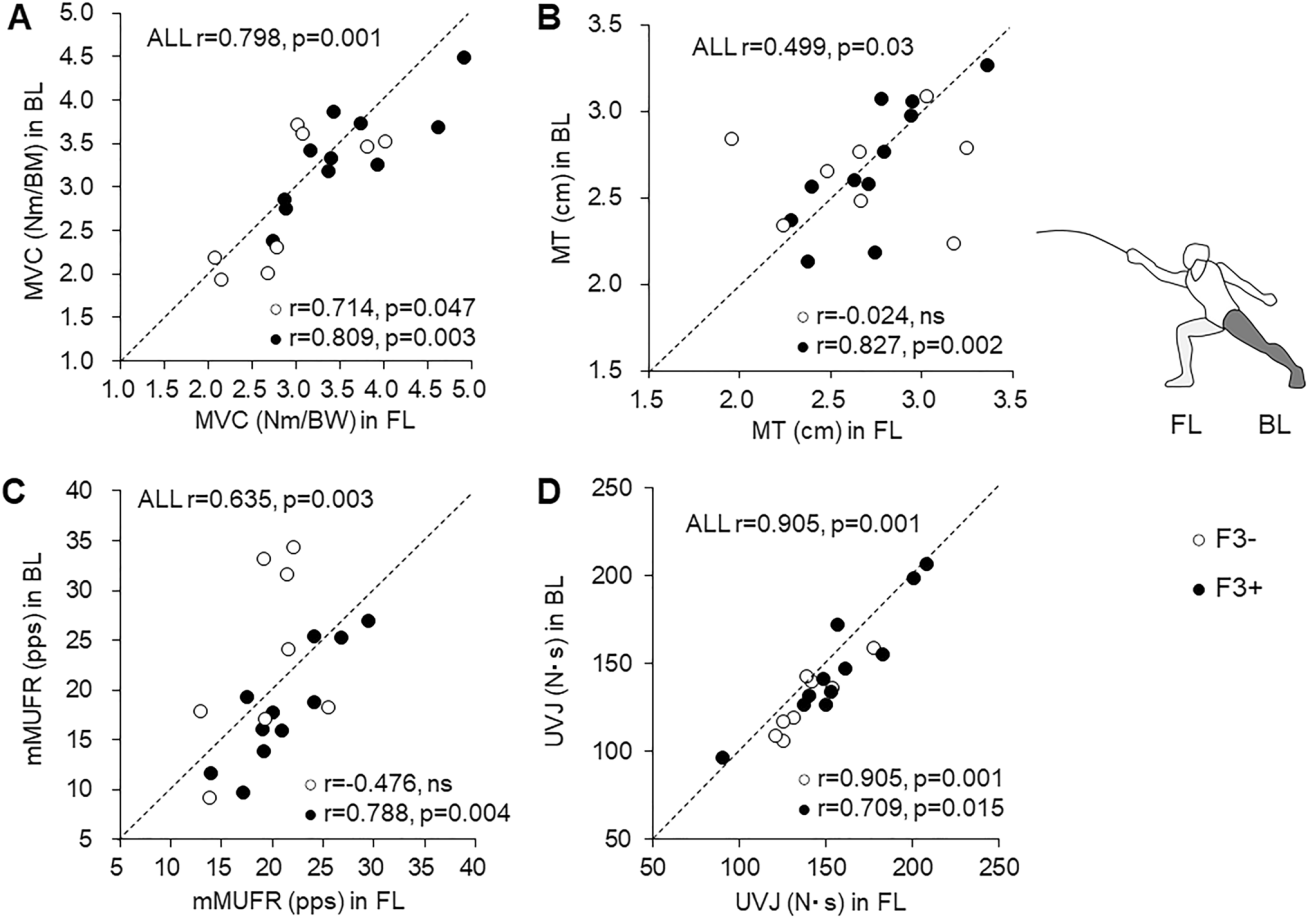
Associations between front leg (FL) and back leg (BL) in maximal voluntary contraction (MVC) (**A**), muscle thickness (MT) (**B**), modified motor unit firing rate (mMUFR) (**C**), and unilateral vertical jump (UVJ) (**D**) for all participants, and fencers with less (F3−) (open circle) and more (F3+) (filled circle) than 3 years of fencing experience

Significant correlations between FL and BL were noted in MVC and UVJ in all participants, F3−, and F3+ (*p* < 0.05) (Fig. 5A, D). There were significant correlations in MT and mMUFR in all participants and F3+ (*p* < 0.05), but not in F3− (*p* > 0.05) (Fig. 5B, C).

For sMUFR, there were no significant differences between FL and BL (*p* > 0.05) and no significant correlations between FL and BL in all participants, F3−, and F3+ (*p* > 0.05).

In the comparisons of sex, MVC and UVJ in FL and BL were significantly greater in male (*p* < 0.05, ES = 0.509 for FL and 0.637 for BL in MVC and 0.531 for FL and 0.531 for BL in UVJ) and sMUFR in BL was significantly greater in female (*p* < 0.05, ES = 0.679). There were no significant differences in MT and mMUFR in both FL and BL and sMUFR in FL (*p* > 0.05). For male, UVJ in FL was significantly greater than BL (*p* < 0.05, ES = 0.578). For female, UVJ in FL was significantly greater than BL (*p* < 0.05, ES = 0.904) and sMUFR in BL was significantly greater than FL (*p* < 0.05, ES = 0.904).

In the comparisons among the weapon types, we did not find any significant differences among foil, epee, and sabre in MVC, MT, mMUFR, sMUFR, and UVJ in FL and BL (*p* > 0.05). Significant greater UVJ in FL than BL was showed in foil (*p* < 0.05, ES = 0.885). There were no significant differences between FL and BL for MVC, MT, mMUFR, and sMUFR in foil, epee, and sabre (*p* > 0.05).

## Discussion

We investigated lateralities of neuromuscular characteristics in legs of junior fencers. Major findings of the present study were: (a) muscle strength and indicators of neural and muscular factors were not significantly different between the legs of junior fencers, and (b) distinct characteristic laterality in dynamic muscle strength and neural factors was found between the junior fencers with less and more than three years of fencing experience. These findings suggest that asymmetries in neuromuscular characteristics reported in elite fencers are not observed in junior fencers, and these characteristics are influenced by the duration of fencing experience.

### Muscle strength

The present study identified no significant differences in variables reflecting static and dynamic muscle strength (MVC and UVJ) between FL and BL for all participants (Fig. 2A, B) (*p* > 0.05). These findings do not support our hypothesis that muscle strength is greater in FL than BL in junior fencers. Nystrom et al. (1990) reported higher isometric and dynamic strength in FL than BL in elite fencers (Nystrom et al. 1990). This laterality in muscle strength has been widely recognized as a physiological characteristic of experienced fencers (Roi and Bianchedi 2008). Since previous studies compared muscle strength between the legs only in those over 20 years of age on average, our results are the first to show symmetric muscle strength in younger fencers, at least to the best of our knowledge. In sub-analysis of the duration of fencing experience, no significant differences in MVC between FL and BL were found in either of the investigated groups (Fig. 3A). However, higher UVJ with FL than BL was noted in the group with shorter fencing history (F3−), but not in the group with longer fencing history (F3+) (Fig. 3D). As the ages of those in F3− and F3+ were matched (Table 1), different results between the groups would be primarily due to variations in the duration of fencing experience. These results indicate that fencing experience exceeding 3 years attenuates asymmetry of dynamic muscle strength. Considering the significant asymmetry of muscle strength in elite fencers with longer experience (Nystrom et al. 1990; Roi and Bianchedi 2008), laterality of legs following fencing experience might develop in two different stages of attenuation and enhancement. Based on the biomechanical data during fencing attacks, FL requires greater joint extension power with eccentric muscle contraction during the braking phase for deceleration of forward body movements than that with concentric muscle contraction in BL during the propulsive phase for acceleration of forward body movements (Chen et al. 2017; Guilhem et al. 2014). Since maximal joint power in FL is approximately 150% of that in BL during fencing attacks, prolonged fencing training would induce greater muscle strength and muscle mass in FL (Guilhem et al. 2014). It is well-known that effects of resistance training on muscle strength are greater in novice compared with trained populations (Douglas et al. 2017; Vogt and Hoppeler 2014). Therefore, we suggest that the significant difference in UVJ only in F3− can be explained by participants’ greater sensitivity to fencing-specific training. The strong positive correlation between FL and BL in UVJ for F3− (Fig. 5D), indicating lower inter-individual differences in asymmetry, but still supporting the asymmetry (for F3−, all BL vs. FL UVJ values are below the diagonal—Fig. 5D), could reflect homogeneous adaptations in early-career fencers and may support our hypothesis. We do not know of any scientific evidence of differences due to training strategies among novice, junior, and elite fencers, but it was considered that an earlier adaptation in FL may occur to maintain the postural range after the attack motion prior to strengthening the joint power to bring BL forward. Also, coaches might design training to minimize asymmetries for junior fencers from a prophylactic point of view (Roi and Bianchedi 2008). Akpinar et al. (2015) reported that fencers exhibit more symmetric performance than non-fencers during arm-reaching movements (Akpinar et al. 2015). While this previous study focused on upper limbs, development of symmetry in physical performance would also occur as an adaptation following fencing training in addition to asymmetric adaptation. This may explain both attenuation and enhancement of laterality in fencers.

A previous study reported that 20–30% of healthy teenagers show a difference of > 15% between limbs in strength and power during UVJ, and the authors concluded that differences of < 15% in UVJ performance between limbs should be considered as the physiological norm in this age group (Ceroni et al. 2012). In the present study, one and two fencers from F3− and F3+, respectively showed a difference of > 15% in UVJ between FL and BL, and FL showed a greater UVJ for all these fencers. Differences in rates > 15% in UVJ were 12.5 and 18.2% in F3− and F3+ in the present study, respectively, indicating that the laterality in UVJ in junior fencers may be physiologically normal.

We found different results among the fencers with different weapon types in UVJ. Greater UVJ in FL than BL was showed in foil but not in epee and sabre. For interpretation of these results, we should note the profiles (sex and/or duration of fencing experience) of fencers with each weapon type. While there are no significant effects of weapon types on duration of fencing experience (*p* > 0.05), all female fencers were categorized to foil. Thus, this characteristic UVJ profile in the fencers with foil may be caused by weapon type and sex. Also, the analyses for different types of weapons were performed with small sample numbers, such as *n* = 10, 4, and 5 for foil, epee, and sabre. To clarify the effects of weapon types, further studies with larger number of sample size are needed.

### Muscle anthropometry

Similar to muscle strength, asymmetries in anthropometry have been recognized as adaptations to fencing-specific lateralized movements (Nystrom et al. 1990; Tsolakis et al. 2006). In addition to elite adult fencers, Tsolakis et al. (2006) reported asymmetries in leg muscle morphology in 14- to 17-year-old groups. Thus, we hypothesized that muscle mass is greater in FL than BL in junior fencers. However, our results for MT did not support this hypothesis. Also, in the report of Tsolakis et al. (2006), asymmetries in leg muscle morphology were not observed in 10- to 13-year-old groups. Since 14- to 17-year-old and 10- to 13-year-old groups had 4.4 and 2.2 years of fencing training experience, respectively, in this previous study (Tsolakis et al. 2006), we also hypothesized that laterality of muscle mass is less pronounced in fencers with a shorter training history. The present study did not detect lateralities in MT in either F3− or F3+ (Fig. 3B), meaning that symmetric leg anthropometry in the present study was independent of the duration of fencing history. However, we found different trends in correlation analysis of MT between F3− and F3+, i.e., significant correlation of MT between FL and BL for F3+, but not for F3− (Fig. 5B). These results suggest that F3− exhibits greater inter-individual variance of laterality in MT and F3+ shows similar trends in laterality within the group. Although we did not identify laterality in MT even in junior fencers with a longer duration of fencing experience, a lower level of inter-individual differences in laterality of muscular components following fencing training was observed. A longer duration would be needed for detectable muscular adaptations.

Although consensus has been reached regarding whether FL in elite fencers shows greater muscle mass, which was reported by computed tomography (Nystrom et al. 1990), limited supporting evidence for that in junior fencers was reported by Tsolakis et al. (2006), who used an anthropometric formula incorporating limb circumference and skin folds (Tsolakis et al. 2006). Ultrasonography used in the present study would also have some methodological limitations for estimation of the muscle mass when compared with computed tomography or magnetic resonance imaging. However, this method should facilitate more direct observation of muscle anthropometry (Takai et al. 2013) than the anthropometric formula incorporating limb circumference and skin folds used by Tsolakis et al. (2006). Also, we measured muscle thickness in longitudinal images which are scanned at the center of surface EMG electrode, i.e., midpoint of the distance between ASIS and lateral patella (Fig. 1). This location would not be standard for assess muscle thickness of knee extensor muscles (Takai et al. 2013). Since variables in ultrasonography images are sensitive to the scanned location, muscle thickness measured at the standard location may provide different results. To clarify laterality in muscle mass or anthropometry in junior fencers, further studies are warranted using other medical imaging techniques.

### Neural factor

We aimed to quantify laterality in central nervous system activation from motor unit firing activation properties. While there was no significant difference in mMUFR between FL and BL for all participants (Fig. 2C), significantly higher mMUFR in FL than BL was observed in F3+, but not in F3− (Fig. 3C). These findings suggest that junior fencers with a longer fencing history show greater neural input from the central nervous system to knee extensor muscles of FL than BL. An increase in the motor unit firing rate is one of the major adaptations following resistance training (Del Vecchio et al. 2019a, b; Gabriel et al. 2006), and well-trained populations like weightlifters show a higher motor unit firing rate (Leong et al. 1999). Therefore, knee extensor muscles in FL would be more adapted than BL in the context of neural components. We also found a significant correlation in F3+ and no significant correlation in F3− for mMUFR between FL and BL, respectively (Fig. 5C). As F3+ demonstrated asymmetry in mMUFR (Fig. 3C), this result of correlation analysis for F3+ suggests that there is less inter-individual variation in laterality within fencers with longer training experience. On the other hand, F3− exhibited larger inter-individual variability in laterality of mMUFR. Greater plasticity in neural than muscular components has been widely recognized (Gabriel et al. 2006; Moritani and deVries 1979; Sale 1988). The common laterality of neural components in F3+ might suggest that neural laterality appears before detectable laterality in muscular components, reported as asymmetric anthropometry in leg muscles of elite fencers (Nystrom et al. 1990). This is in agreement with our novel concept regarding the development of asymmetric muscle strength following fencing experience with both attenuation and enhancement stages. Greater neural adaptation in FL in junior fencers may be a predictor of fencing-specific asymmetries in muscle strength and anthropometry.

Although we interpreted an increase in motor unit firing rate as neural adaptations to physical training based on the results of the previous studies (Del Vecchio, et al. 2019a, b; Gabriel et al. 2006; Leong et al. 1999), some other studies including our previous works reported unchanged or decreased motor unit firing rate following resistance training (Kidgell et al. 2006; MacLennan et al. 2022; Pucci et al. 2006; Rich and Cafarelli 2000; Sterczala et al. 2020; Watanabe et al. 2018a, b; Watanabe et al. 2020). While we believe that an increase in motor unit firing rate reflects greater neural input from the central nervous system to peripheral muscles (Del Vecchio et al. 2019a, b), adaptations in motor unit firing properties may be complicated processes. This could explain that greater motor unit firing rate in FL for F3+ (Fig. 3C) is not reflected in MVC or UVJ (Fig. 3A, D). We thus think that additional measurements, which can quantify neural activities, or other detailed analyses of motor unit firing pattern such as discharge synchronization (Del Vecchio et al. 2020; Kidgell et al. 2006) could help to characterize neural components of athletic performance.

Relationship between sex-differences in muscle size and motor unit properties have been reported (Trevino et al. 2019), suggesting that male and female show different characteristics not only in muscle components but also in neural components in athletic performance. The present study showed sex-difference in motor unit firing properties in addition to muscle strength and jump performance, such as greater sMUFR for BL in female than male. While effect size was not low (0.679), sample size for comparisons between sex was small (5 females) in the present study. Future study should try to increase sample number of female participants to identify the sex-differences in neuromuscular system for FL and BL in fencers.

## Limitations

We only measured the VL muscle, which is one of the knee extensor muscles, for both neural and muscular factors. Other muscle components in knee extensors or other leg muscle groups, such as hamstrings, may show different neuromuscular adaptations between limbs. We should state that the results of the present study are observed only from the VL muscle and it is unclear whether the results of the present study can apply to other muscles or muscle groups.

In the present study, we analyzed approximately 7 (3–16) motor units for one leg in individual fencers to characterize individual differences in neural activation. Because we analyzed motor unit firing properties during relatively higher force production, the motor units recruited from low to high force levels could be detected. However, it is difficult to think that motor units detected in the present study reflect motor unit firing properties of whole muscles, since surface EMG can detect motor unit action potentials from superficial area of the muscles (Fuglevand et al. 1992). Analyses of larger number of motor units might provide the detailed knowledge for neural activation.

## Conclusion

The present study investigated leg laterality of neuromuscular characteristics in junior fencers, and the effects of the duration of fencing experience on them were also examined. Muscle strength and indicators of neural and muscular factors were not significantly different between the legs. Characteristic laterality in dynamic muscle strength and neural factors was noted between the fencers with less and more than 3 years of fencing experience. From these findings, we suggest that there are no detectable asymmetries in muscle strength, muscle anthropometry, or neural activation in junior fencers. Also, differences in the duration of fencing experience induce characteristic laterality in neural activation and dynamic muscle strength in junior fencers.
